# The Betel Nut

**Published:** 1873-07

**Authors:** 


### THE BETEL-NUT.

There is a fascination in the betel-nut more extraordinary than in the tobacco passion. The consumption of the latter in chewing alone, in the United States, is a modern phenomenon. An inveterate chewer may have moral resolution enough to break off the habit, though it rarely happens that an effort is made to do so, as an apology is found for continuing a practice that is positively destroying the foundations of health. Once addicted to chewing tobacco, to abandon it is an achievment few have the happiness to perform, notwithstanding the melancholy mortality of men in the meridian of life who are constantly being destroyed by the subtle influence of that strange plant on the nervous system. Thus sudden palsy of the heart, palsy of a limb, palsy of one-half the tongue, and even instantaneous death, are traceable by physicians to excessive use of tobacco. But the vice of betel-nut chewing is still more remarkable. When the habit is established there seems no retreat. The victim wears out his teeth, gums, and digestion, and dies with an unsatisfied longing for another quid. Betel-nut trees thrive in most parts of tropical India, the Indian Archipelago, and the Philippine Islands. They grow up gracefully about thirty feet, rarely more than eight inches in diameter. It is an areca catechu. Penang is the universal name of the nut in those places where it is produced; hence, Pulo Penang means betel-nut island. At six years of age the tree commences bearing nuts of the size of a small pullet's egg, of a bright yellow color, inclosed in husk similar to that of a cocoa-nut; within is a spherical nut, very much like a nutmeg.—Broken, a bit of it is wrapped up with a piece of unslacked lime in a peculiar leaf, the Siri betelpiper, extensively cultivated for that purpose. The gums and mucous membrane of the mouth are quickly stained a brick red; the teeth crumble to a level with the gums; and in that condition an inveterate betel-chewer is wretched without a supply. There are large plantations of betel-nut trees in Java to meet the demand for home consumption and in distant provinces. To augment the pleasure, those who can afford it add tobacco to the lime.—*Harper's Weekly.*

